# Mitochondrial Oxidative Stress Promotes Cardiac Remodeling in Myocardial Infarction through the Activation of Endoplasmic Reticulum Stress

**DOI:** 10.3390/antiox11071232

**Published:** 2022-06-23

**Authors:** Francisco V. Souza-Neto, Fabian Islas, Sara Jiménez-González, María Luaces, Bunty Ramchandani, Ana Romero-Miranda, Beatriz Delgado-Valero, Elena Roldan-Molina, Melchor Saiz-Pardo, Mª Ángeles Cerón-Nieto, Luis Ortega-Medina, Ernesto Martínez-Martínez, Victoria Cachofeiro

**Affiliations:** 1 Departamento de Fisiología, Facultad de Medicina, Instituto de Investigación Sanitaria Gregorio Marañón (IiSGM), Universidad Complutense de Madrid, 28040 Madrid, Spain; franvasc@ucm.es (F.V.S.-N.); saraji02@ucm.es (S.J.-G.); anarom12@ucm.es (A.R.-M.); beadel02@ucm.es (B.D.-V.); 2 Servicio de Cardiología, Instituto Cardiovascular, Hospital Clínico San Carlos, 28040 Madrid, Spain; fabianislas@salud.madrid.org (F.I.); mluaces@salud.madrid.org (M.L.); 3 Servicio de Cirugía Cardiaca Infantil, Hospital La Paz, 28046 Madrid, Spain; bunty.r@salud.madrid.org; 4 Biobanco del Hospital Clínico San Carlos, Instituto de Investigación de Salud del Hospital Clínico San Carlos, 28040 Madrid, Spain; elenamilagrosa.molina@salud.madrid.org (E.R.-M.); luis.ortega@salud.madrid.org (L.O.-M.); 5 Departamento de Patología, Hospital Clínico San Carlos, 28040 Madrid, Spain; melchor.saiz@salud.madrid.org (M.S.-P.); nines.ceron@salud.madrid.org (M.Á.C.-N.); 6 Departamento de Medicina Legal, Psiquiatría y Patología, Universidad Complutense de Madrid, 28040 Madrid, Spain; 7 Ciber de Enfermedades Cardiovasculares (CIBERCV), Instituto de Salud Carlos III, 28222 Majadahonda, Spain

**Keywords:** cardiac fibrosis, mitochondrial oxidative stress, endoplasmic reticulum stress, myocardial ischemia

## Abstract

We have evaluated cardiac function and fibrosis in infarcted male Wistar rats treated with MitoQ (50 mg/kg/day) or vehicle for 4 weeks. A cohort of patients admitted with a first episode of acute MI were also analyzed with cardiac magnetic resonance and T1 mapping during admission and at a 12-month follow-up. Infarcted animals presented cardiac hypertrophy and a reduction in the left ventricular ejection fraction (LVEF) and E- and A-waves (E/A) ratio when compared to controls. Myocardial infarction (MI) rats also showed cardiac fibrosis and endoplasmic reticulum (ER) stress activation. Binding immunoglobulin protein (BiP) levels, a marker of ER stress, were correlated with collagen I levels. MitoQ reduced oxidative stress and prevented all these changes without affecting the infarct size. The LVEF and E/A ratio in patients with MI were 57.6 ± 7.9% and 0.96 ± 0.34, respectively. No major changes in cardiac function, extracellular volume fraction (ECV), or LV mass were observed at follow-up. Interestingly, the myeloperoxidase (MPO) levels were associated with the ECV in basal conditions. BiP staining and collagen content were also higher in cardiac samples from autopsies of patients who had suffered an MI than in those who had died from other causes. These results show the interactions between mitochondrial oxidative stress and ER stress, which can result in the development of diffuse fibrosis in the context of MI.

## 1. Introduction

Cardiomyocyte necrosis, granulation tissue formation, inflammation, and scar maturation are important events in the healing process after myocardial infarction (MI) [1]. The loss of functional myocardium results in a cardiac remodeling in which fibrosis plays a central role in the structural changes in the left ventricle (LV) [2,3] as well as in energy metabolism alterations and impaired myocardial perfusion. This cardiac remodeling is produced in the infarcted border zone and remote non-infarcted myocardium, where the accumulation of fibrosis can result in functional consequences [4,5]. All these alterations can attenuate diastolic and systolic functions, producing a significant impact on the quality and quantity of life.

Multiple mechanisms have been proposed as potential players in cardiac remodeling in the context of MI, including oxidative stress. MI triggers excessive reactive oxygen species (ROS) production, especially in response to the reperfusion that is produced in subcellular organelles such as the sarcolemma, endoplasmic reticulum (ER), and especially mitochondria through the electron transport chain [6]. This oxidant environment produces damage to the mitochondria themselves, further facilitating mitochondrial dysfunction. The heart is the most metabolically active organ in the body, which possesses the highest mitochondria content of any tissue and obtains its energy primarily from these. Mitochondrial dysfunction produces cellular energy disruption and oxidative stress, which triggers cardiac cell loss after damage caused by a compromised supply of the energy necessary for reaching the intense demands of the heart [7,8].

The ER is a dynamic cellular organelle with different functions, including Ca^2+^ buffering and protein synthesis, folding, and processing, among others [9,10]. In a physiological state, the folding capacity of the ER is not saturated because protein synthesis and folding levels follow a balance. However, in response to excessive oxidative stress, misfolded proteins accumulate in the ER lumen, promoting ER stress and the activation of the unfolded protein response (UPR) in order to normalize and return the balance [10]. This response involves the dissociation of binding immunoglobulin protein (BiP) from three transmembrane proteins. Consequently, an upregulation of BiP is considered to be a marker of ER stress activation [11]. However, it could promote inflammation, cell dysfunction, death, and tissue injury if ER stress is not resolved. ER stress activation is associated with myocardial fibrosis due to its ability to promote fibroblast differentiation to myofibroblasts with an enhanced proliferation capacity and higher synthesis of extracellular matrix (ECM) components [12]. Both the ER and mitochondria can come into contact, thereby forming the mitochondria–ER-associated membranes through which they can coordinate their functions by participating in the regulation of different processes. However, whether these interactions can be relevant for the development of cardiovascular fibrosis in the context of MI is not fully established. Therefore, the aim of this study was to evaluate whether the crosstalk between mitochondrial oxidative stress and ER stress activation is relevant in the development of cardiac remodeling in the context of MI. Accordingly, we evaluated the effect of the mitochondrial-targeted antioxidant MitoQ in cardiac fibrosis in MI rats. Additionally, we studied how MitoQ treatment can affect ER stress activation in this pathological context. We also explored the potential relationship between cardiac fibrosis and the activation of ER stress in patients with MI.

## 2. Materials and Methods

### 2.1. Animal Model

MI was induced by ligation of the left anterior descendent (LAD) coronary artery in 12-week-old male Wistar rats (Envigo, Barcelona, Spain; *n* = 20 rats), as previously described [5]. A group of animals subjected to a sham operation (the same surgical procedure without the fastening of the suture that passes through the LAD; *n* = 20 rats) was included as a reference group (CT). Both groups of animals received the mitochondrial antioxidant MitoQ (50 mg/kg/day, equivalent to 200 µM) in the drinking water for 4 weeks after surgery. The dose of MitoQ was based on previous data [13]. MitoQ was provided by MP Murphy from the Medical Research Council Mitochondrial Biology Unit, Cambridge BioMedical Campus, Cambridge, UK. The body weight, systolic blood pressure (SBP), and cardiac structure and function were measured at the end of the evolution period. The SBP was estimated through use of a tail-cuff plethysmograph (Cibertec, Madrid, Spain) in unrestrained rats. At the end of the experiment, serum and plasma were collected. The Animal Care and Use Committee of Universidad Complutense de Madrid and Dirección General de Medio Ambiente, Comunidad de Madrid, Spain, approved all experimental procedures (PROEX 121/18).

### 2.2. Cardiac Function Measurement

Cardiac function was evaluated by transthoracic echocardiography with a Vivid-I (General Electric Healthcare, Boston, MA, USA) connected to a 12 MHz transducer. Then, 2D-guided M-mode recordings were made from short-axis views at the level of the papillary muscles to measure the left ventricular (LV) chamber end-diastolic dimensions, interventricular septum, and posterior wall thickness. The mean measurements from several consecutive beats were used for the data analysis. The end-diastolic diameter (EDD) and end-systolic diameter (EDS) were used to calculate the left ventricular ejection fraction (LVEF) and the fractional shortening (FS). The LVEF was calculated in percentage according to the Teicholz formula: (EDD^3^ × 7)/(2.4 + EDD), and the LV systolic chamber function was determined from the LV endocardial FS. The FS was calculated in percentage according to the formula: (EDD−EDS)/EDD. The diastolic function was assessed by the early and late transmitral peak diastolic flow velocity (E and A waves), and the ratio between E-waves and A-waves (E/A) was calculated. The images were processed with the Echopack software v. 201 (General Electric Healthcare, Boston, MA, USA).

The LV mass and infarct size were defined by magnetic resonance imaging (MRI). Both the LV mass and myocardial deformation parameters such as the radial strain and circumferential strain were analyzed in basal, medial, and apical short axis slices from steady state free precession images using a 17-segment model. The MRI study was performed with a Biospec BMT 47/40 spectrometer (Bruker, Ettlingen, Germany) located at the NMR Center of the Universidad Complutense of Madrid that was equipped with a 12 cm gradient system and connected to a 1025 SAM monitoring and a gating system (SA Instruments, Inc., New York, NY, USA), as is previously described [5]. The images were processed with the MEDIS SUITE MR software (Medis Medical Imaging Systems, Leiden, The Netherlands).

### 2.3. Morphological and Histological Evaluation

Cardiac tissue samples were dehydrated, embedded in paraffin, and cut in 4 μm thick sections. The sections were stained with picrosirius red in order to detect collagen fibers. The area of cardiac interstitial fibrosis was identified as the ratio of interstitial fibrosis or collagen deposition to the total tissue area after excluding the vessel area from the region of interest. For each sample, 10 to 15 fields were analyzed with a 40× objective under transmitted light microscopy (Leica DM 2000; Leica AG, Wetzlar, Germany). The cross-sectional area was measured in myocytes along the left ventricle (60–80 per animal) with visible nuclei, and intact cellular membranes were chosen for the determination of the cross-sectional area in hematoxylin and eosin staining sections with a 40× objective under transmitted light microscopy (Leica DM 2000) in three different sections. A quantitative analysis were performed using an analysis system (Leica LAS 4.3; Leica AG, Wetzlar, Germany).

### 2.4. Measurement of Cardiac Reactive Oxygen Species (ROS) Levels

For the detection of total superoxide anion (O^2−^) levels, cardiac sections (14 µm) were incubated with dye dihydroethidium (DHE; 10^−5^ M) for 30 min at 37 °C. DHE is oxidized by superoxide and exhibits red fluorescence. The oxidation products become highly fluorescent upon binding to nucleic acids. The fluorescent signals were viewed using a fluorescent laser scanning microscope (40× objective in a Leica DMI 3000 microscope; Leica AG, Wetzlar, Germany). The quantitative analysis of O^2−^ production was performed using an image analyzer (Leica LAS 4.3; Leica AG, Wetzlar, Germany). Three sections per animal were quantified and averaged for each experimental condition. The mean fluorescence densities in the target region were analyzed. The results are expressed as n-fold increases over the values of the control group in arbitrary units.

### 2.5. Western Blot Analysis

Total cardiac proteins from the non-infarcted area were separated by SDS-PAGE on 4–15% polyacrylamide gels and transferred to Hybond-c Extra nitrocellulose membranes (Hybond-P; Amersham Biosciences, Piscataway, NJ, USA) with the Trans-Blot Turbo Transfer System. Membranes were probed with primary antibodies for activating transcription factor 4 (ATF4; Proteintech, Rosemont, IL, USA; dilution:1:1000; Ref: 10835-1-AP), activating transcription factor 6 alpha, (ATF6α; Santa Cruz, Dallas, TX, USA; dilution: 1:250; Ref: sc-166659), binding immunoglobulin protein (BIP; BD Biosciences, Madrid, Spain; dilution 1:1000; Ref: 610978), calpain 1 (Abcam, Cambridge, UK; dilution 1:1000; Ref: ab39170), calreticulin (CALRT; Cambridge, UK; dilution 1:1000; Ref: ab92516), CCAAT-enhancer-binding protein homologous protein (CHOP; Cell Signaling Technology, Danvers, MA, USA; dilution 1:500; Ref: #2895), collagen I (Calbiochem, San Diego, CA, USA; dilution 1:1000; Ref: 234167), connective tissue growth factor (CTGF; Sigma-Aldrich, Cambridge, UK; dilution 1:1000; Ref: C4871), cyclophilin F (Santa Cruz Biotechnology; Dallas, TX, USA; dilution 1:500; Ref: Sc-376061), mitofusin 1 (MFN1, Abcam; Cambridge, UK; dilution 1:1000; Ref: ab126575), transforming growth factor β (TGF β; Abcam, Cambridge, UK; dilution 1:500; Ref: ab190503), and glyceraldehyde 3-phosphate dehydrogenase (GAPDH; Cell Signaling; dilution: 1:5000; Ref: #5174) as a loading control. The signals were detected using the ECL system (Millipore, Burlington, MA, USA). Several proteins were analyzed in the same membrane after a stripping procedure (Thermo Scientific, Waltham, MA, USA; Ref: 21063). The results are expressed as n-fold increases over the values of the control group in arbitrary densitometric units.

### 2.6. Clinical Study

A cohort of 44 patients admitted to a tertiary care center (Hospital Clínico San Carlos, Madrid, Spain) with a first episode of acute MI were studied. MI was defined by “The Third Universal Definition of Myocardial Infarction” [14]: an increase in the biomarkers in the presence of ischemia, ST-segment -T wave (ST-T) changes, the appearance of new Q waves, the identification of alterations in local contraction by imaging techniques, or intracoronary thrombus detected by angiography. Twenty-four to forty-eight hours after hospital admission for MI and 12 months after follow-up, patients underwent transthoracic echocardiography, and, at the same time, blood samples were collected. The study protocol was approved by the ethics committee (18/195-E), and all participants signed the informed consent. The present study was conducted in compliance with the good clinical practice guidelines and the ethical principles stated in the Declaration of Helsinki.

### 2.7. Cardiac Function

Transthoracic echocardiographic (TTE) images were acquired according to the cavity quantification guidelines of the American Society of Echocardiography and the European Society of Cardiovascular Imaging [15]. Conventional TTE planes were obtained, such as the parasternal long axis, in which the left ventricle (LV) wall thickness was measured for ventricular mass calculation and classification. In addition, two-chamber (2C), three-chamber (3C), and four-chamber (4C) apical planes were obtained, and the LV end-diastolic (EDV) and end-systolic (ESV) volumes were measured.

The LVEF was calculated from the quantification of the LVEDV and LVESV obtained in the apical 4C and apical 2C planes using the following formula: LVEF = (LVEDV – LVESV)/LVEDV. Diastolic function was assessed according to the current guideline recommendations as well. Diastolic function was assessed by the early and late transmitral peak diastolic flow velocity (E and A waves), and the ratio between the E-waves and A-waves (E/A) was calculated. The images were processed with the Echopack software v.201(General Electric Healthcare, Boston, MA, USA).

### 2.8. Myocardial Extracellular Volume Fraction

T1- and T2-weighted sequences were included in the study to detect late gadolinium enhancement (LGE) and edema. Quantitative T1 mapping was performed with modified look-locker inversion-recovery (MOLLI) sequences for the identification and quantification of the extracellular volume in the affected territory and in the territory remote to the infarct [16,17]. The analysis of the cardiac MRI images was performed with Medis Suite MR (Medis Medical Imaging Systems, Leiden, The Netherlands).

### 2.9. Circulating Plasma Levels of Myeloperoxidase (MPO) and BiP

Circulating MPO and BiP levels were measured by sensitive enzyme immunoassays (R&D Systems, Minneapolis, MN, USA; Ref:DY3174 and Abbexa, Cambridge, UK; Ref: abx251110, respectively) following the instructions of the manufacturers.

### 2.10. Analysis of Biobank Cardiac Samples. Cardiac Immunohistochemistry of BiP

BiP immunohistochemical staining was measured in LV myocardial tissue obtained from patients who had suffered MI (*n* = 14) from the Hospital Clínico San Carlos biobank. Patients that had died from another cause were used as controls (*n* = 14). Immunostaining was detected using a rabbit polyclonal antibody (Abcam, Cambridge; UK, dilution 1:100) in 4 μm heart sections in samples fixed in buffered formaldehyde and paraffin-embedded using an automated method (Dako Autostainer). After counterstaining with hematoxylin, slides were dehydrated in ascending concentrations of ethanol and mounted. An external negative control was included. A blinded staining evaluation was performed by an experienced pathologist who did not have access to the clinicopathological data. For each sample, 25 fields were analyzed with a 20× objective under a transmitted light microscope (Leica DM 2000; Leica AG, Wetzlar, Germany). The area of BiP staining was identified as the ratio of staining to the total tissue area after excluding either the necrotic or vessel areas from the region of interest. The quantitative analysis was performed using an analysis system (Leica LAS 4.3; Leica AG, Wetzlar, Germany). This study was approved by the Medical Ethics Review Committee of Hospital Clínico San Carlos (21/168-E).

### 2.11. Cardiac Collagen Content

Cardiac sections from autopsies from the Hospital Clínico San Carlos biobank were dehydrated, embedded in paraffin, and cut in 4 μm thick sections. The samples were stained with picrosirius red in order to detect collagen fibers. The area of cardiac interstitial fibrosis was identified as the ratio of interstitial fibrosis or collagen deposition to the total tissue area after excluding the vessel area from the region of interest. For each sample, 25 fields were analyzed with a 40× objective under a transmitted light microscope (Leica DM 2000; Leica AG, Wetzlar, Germany). The quantitative analysis was performed using an analysis system (Leica LAS 4.3; Leica AG, Wetzlar, Germany).

### 2.12. Statistical Analysis

Continuous variables are expressed as means ± standard deviation (SD). Categorical variables are expressed in absolute values and percentages. The differences between categorical variables were analyzed using the chi-square test. The normality of distributions was verified by means of the Kolmogorov–Smirnov test. In the case of continuous variables, differences between two groups were analyzed by either an unpaired Student’s *t*-test test or a Mann–Whitney test as the parametric and non-parametric tests, respectively. Specific differences between more groups were analyzed using Kruskall–Wallis tests followed by Dunn’s test for variables with non-normal distributions. For variables with normal distributions, a one-way analysis of variance was used followed by a Newman–Keuls test. Either Pearson or Spearman correlation analyses were used to examine the associations among different variables according to whether they were normally distributed. A value of *p* < 0.05 was used as the cut-off value for defining statistical significance. The data analysis was performed using the statistical programs GraphPad Prism 8 (San Diego, CA, USA) and SPSS version 25.0 (SPSS Inc., Chicago, IL, USA).

## 3. Results

### 3.1. Animal Study

**Figure 1:**
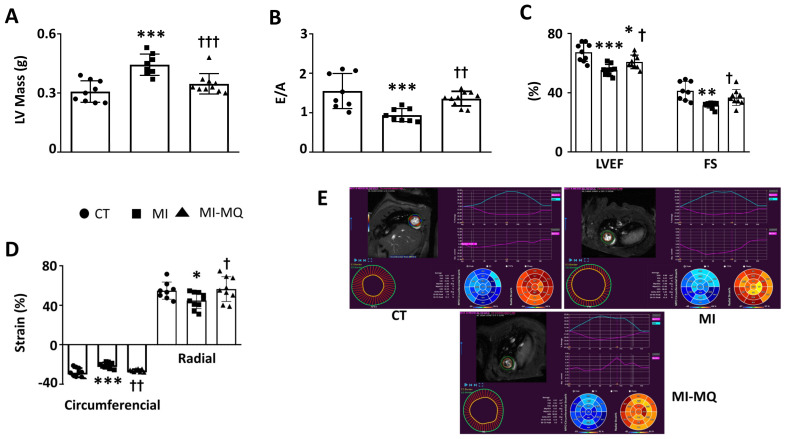
Mitochondrial oxidative stress mediates cardiac structural and functional alterations in infarcted rats. (**A**) Left ventricle mass; (**B**) E-wave and A-wave ratio (E/A); (**C**) left ventricle ejection fraction (LVEF) and fractional shortening (FS); (**D**) circumferential and radial strain; (**E**) representative image depicting myocardial segmentation and data analysis for global circumferential and radial strain evaluation using feature-tracking CMR imaging in control rats (CT) and rats submitted to myocardial infarction treated with vehicle (MI) or with the mitochondrial antioxidant MitoQ (MI-MQ; 50 mg/kg/day). Bar graphs represent the means ± SD of 8-10 animals. * *p* < 0.05, ** *p* < 0.01, *** *p* < 0.001 vs. CT group. † *p* < 0.05, †† *p* < 0.01, ††† *p* < 0.001 vs. MI group.

Body weight and blood pressure were similar in all groups (Table S1). However, MI was associated with increases in the cardiomyocyte cross-sectional area (Figure S1A), relative heart weight (Figure S1B), and left ventricle mass (Figure 1A), suggesting the presence of cardiac hypertrophy. No significant differences were observed in the thickness of either the interventricular septum (IVST) or posterior wall (PWT) between the CT and MI animals (Figure S2A,C). No changes were observed in EDD among any group, although an increase in ESD was observed in the animals with MI, which was prevented by MitoQ (Figure S2B,C). MI rats also showed functional alterations: reductions in the E/A ratio, LVEF, and SF (Figure 1B,C and Figure S3). Moreover, infarcted rats showed a reduction in the circumferential and radial strain (Figure 1D,E). The inhibition of mitochondrial oxidative stress by MitoQ was able to prevent all these changes without any effect on the size of the infarcted area (Table S1). The MitoQ treatment did not affect cardiac function or any other evaluated parameter in the control animals (Figure S4). Therefore, and to simplify the results, only CT and MI rats treated with vehicle or MitoQ (MI-MQ) will be considered here.

**Figure 2:**
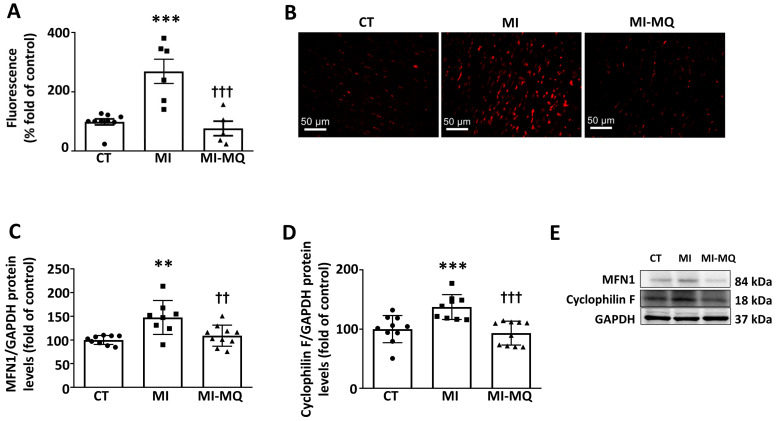
Effect of a mitochondrial antioxidant on superoxide anion levels and mitochondrial protein expression in infarcted rats. (**A**) Quantification of cardiac superoxide anion levels and (**B**) representative microphotographs of cardiac sections labelled with the oxidative dye dihydroethidium (magnification 40×). Protein levels of (**C**) mitofusin 1 (MFN1) and (**D**) cyclophilin F and (**E**) representative blots for protein expression in cardiac tissue from control rats (CT) and rats submitted to myocardial infarction treated with vehicle (MI) or with the mitochondrial antioxidant MitoQ (MI-MQ; 50 mg/kg/day). Bars graphs represent the means ± SD of 8–10 animals, normalized for glyceraldehyde 3-phosphate dehydrogenase (GAPDH). ** *p* < 0.01, *** *p* < 0.001 vs. CT group. †† *p* < 0.01, ††† *p* < 0.001 vs. MI group.

MI produced an oxidant environment characterized by an increase in superoxide anion levels (Figure 2A,B) that was prevented by MitoQ, supporting the effectiveness of the treatment (Figure 2A,B). In addition, MitoQ was able to prevent the mitochondrial alterations observed in MI rats, such as the upregulation in mitofusin 1(MFN1) and cyclophilin F levels (Figure 2C,D). Figure S5 shows the original blots corresponding to Figure 2E.

**Figure 3:**
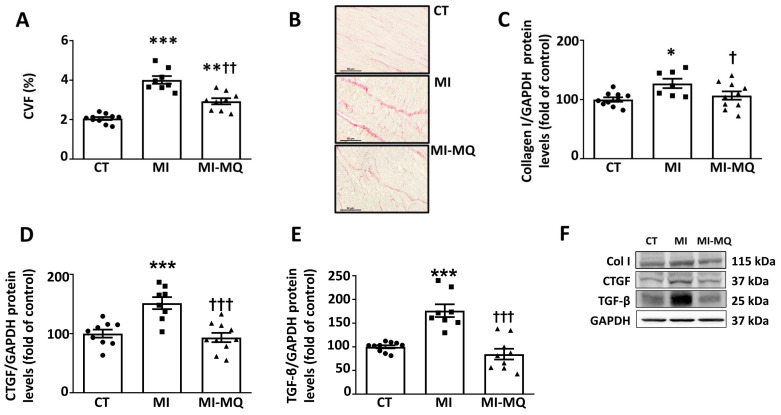
Mitochondrial oxidative stress mediates cardiac fibrosis in infarcted rats. (**A**) Quantification of collagen volume fraction (CVF); (**B**) representative microphotographs of cardiac sections stained with picrosirius red (magnification 40×). Protein levels of (**C**) collagen type I (Col I), (**D**) connective tissue growth factor (CTGF), and (**E**) transforming growth factor-beta (TGF-β) and (**F**) representative blots for protein expression in cardiac tissue from control rats (CT) and rats submitted to myocardial infarction treated with vehicle (MI) or with the mitochondrial antioxidant MitoQ (MI-MQ; 50 mg/kg/day). Bars graphs represent the means ± SD of 8–10 animals, normalized for glyceraldehyde 3-phosphate dehydrogenase (GAPDH). * *p* < 0.05, ** *p* < 0.01, *** *p* < 0.001 vs. CT group. † *p* < 0.05, †† *p* < 0.01, ††† *p* < 0.001 vs. MI group.

As expected, MI was associated with diffuse fibrosis (Figure 3A,B) in the LV, which was positively correlated with cardiac oxidative stress levels (r = 0.7263; *p* = 0.0022) and ameliorated with MitoQ (Figure 3A,B). The hearts from infarcted rats showed an increase in the protein levels of collagen I, the main component of ECM at the cardiac level. An increase was also observed in the protein levels of the ECM mediators connective tissue growth factor (CTGF) and transforming growth factor β (TGF-β) in MI animals (Figure 3C–E). The upregulation of all these proteins was prevented in the animals treated with MitoQ (Figure 3C–E). Figure S5 shows the original blots corresponding to Figure 3F.

**Figure 4:**
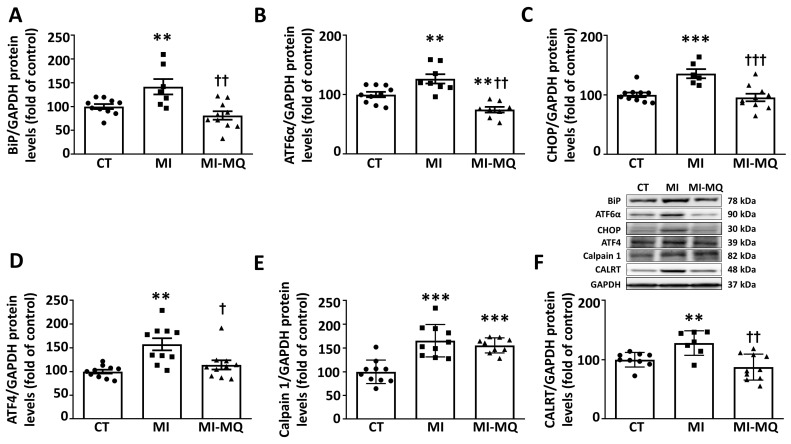
Mitochondrial oxidative stress mediates the cardiac activation of endoplasmic reticulum stress in infarcted rats. Protein levels of (**A**) immunoglobin binding protein (BiP); (**B**) activating transcription factor 6 alpha (ATF6α); (**C**) CCAAT-enhancer-binding protein homologous protein (CHOP); (**D**) activating transcription factor 4 (ATF4); (**E**) calpain 1; and (**F**) calreticulin (CALRT) in cardiac tissue from control rats (CT) and rats submitted to myocardial infarction treated with vehicle (MI) or with the mitochondrial antioxidant MitoQ (MI-MQ; 50 mg/kg/day). Bars graphs represent the means ± SD of 8–10 animals, normalized for glyceraldehyde 3-phosphate dehydrogenase (GAPDH). ** *p* < 0.01, *** *p* < 0.001 vs. CT group. † *p* < 0.05, †† *p* < 0.01, ††† *p* < 0.001 vs. MI group.

MI was also accompanied by cardiac ER stress activation, as suggested by an increase in an ER stress marker, BiP (Figure 4A). These levels were associated with those of either fibrosis (r = 0.7022; *p* = 0.0024) or collagen I (r = 0.7461; *p* = 0.0014). Complementary analyses of different pathways involved in ER stress activation revealed (ATF6α, CHOP, and ATF4 (Figure 4B–D) upregulation in the hearts of infarcted animals. Treatment with MitoQ was able to prevent ER stress activation as well as the increase in ATF6α, CHOP, and ATF4 levels (Figure 4A–D). Infarcted rats showed increases in the cardiac levels of both calpain 1 and CALRT. The MitoQ treatment was only able to prevent the increase in CALRT (Figure 4E,F). Figure S5 shows the original blots corresponding to Figure 4F.

### 3.2. Clinical Study

The mean age of patients was 57.5 ± 10.1 years, and 92.7% were male. The majority (95.1%) of patients had associated comorbidities, with the most frequent being overweight or obesity (78%), hypertension (36.6%), dyslipidemia (36.7%), and diabetes mellitus (22.0%). Drug treatment for hypertension included angiotensin converting enzyme (ACE) inhibitors or angiotensin II type 1 receptor antagonists, whereas all dyslipidemic patients were on statins. After the MI, 80% of patients were on beta-blockers, 87.5% were on ACE inhibitors or angiotensin II type 1 receptor antagonists, and 100% were on statins and antiplatelet therapy. All patients underwent a primary percutaneous intervention during admission, and a culprit lesion was treated in all cases with drug-eluting stents. The anterior descending coronary artery was responsible in 61% of cases. The echocardiography findings of patients are shown in Table 1 in basal conditions and after a 12-month follow-up. Patients with MI showed reduced LVEF and E/A ratio values after hospital admission for MI (Table 1). No major changes in cardiac function were observed in the 12-month follow-up (Table 1). However, patients showed reductions in IVST (*p* = 0.046), PWT (*p* < 0.0002), and EDD (*p* = 0.0458) (Table 1) at 12 months after MI.

**Figure 5:**
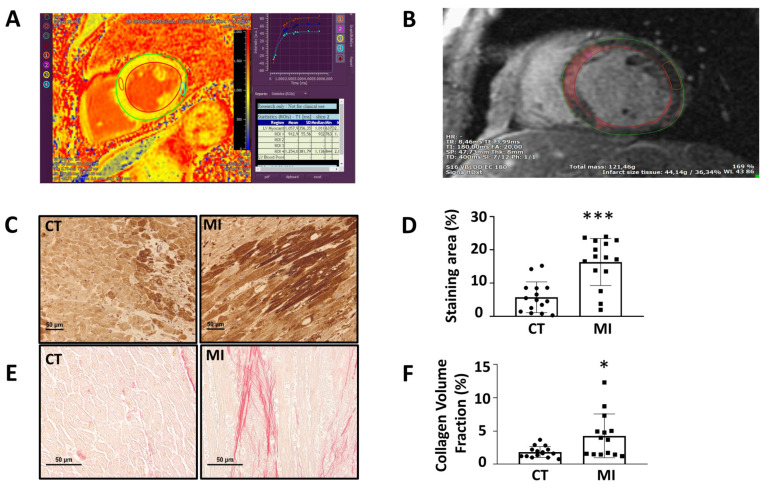
(**A**) A representative image of T1 map with MOLLI sequences of a patient with acute myocardial infarction; (**B**) a representative image of LGE quantification. The mean signal intensity was determined by drawing a region of interest (ROI) in a portion of the normal myocardium, and for comparison, a threshold technique was performed using 5 SDs above the mean signal intensity for the normal nulled myocardium. (**C**) Representative microphotographs of cardiac sections of BiP staining from patients with (MI) or without myocardial infarction (CT); (magnification 20×); (**D**) quantification of BiP staining in cardiac sections from patients with or without myocardial infarction; (**E**) representative microphotographs of cardiac sections staining with picrosirius red of patients with or without myocardial infarction (magnification 40×). (**F**) Quantification of collagen volume fraction (CVF) in cardiac sections of patients with or without myocardial infarction. * *p* < 0.05, *** *p* < 0.001 vs. CT group.

Figure 5A,B illustrate representative native T1 and late gadolinium enhancement (LGE) imaging, respectively, in patients with acute MI. It can be appreciated that the areas of signal hyperenhancement in LGE correspond to elevated extracellular volume fractions (ECV).

No changes were found in the follow-up in either the myocardial ECV or LV mass (Table 1), although a significant reduction was observed in the infarcted mass (*p* = 0.0006). Interestingly, a marker of oxidative stress, myeloperoxidase (MPO), was increased after the 12-month follow-up (*p* = 0.0014). It is noteworthy that patients with BMI > 25 had higher levels of MPO at follow-up (81.58 ± 60.76 vs. 57.51 ± 40.62 ng/mL; *p* = 0.342) and significantly lower LVEF relative to patients with BMI < 25 kg/m^2^ (57.4 ± 7.4 vs. 64.7 ± 5.4%; *p* = 0.009). In addition, MPO levels were associated with myocardial ECV in basal conditions (r = 0.375; *p* = 0.05), showing the possible relevance of oxidative stress to ECM production. Circulating BiP levels were not detected in either the basal conditions or in the 12-month follow-up.

### 3.3. Analysis of Biobank Cardiac Samples

Taking into consideration that circulating BiP levels were undetected in patients, we analyzed BiP staining in cardiac sections from autopsies obtained from the Hospital Clínico San Carlos biobank. Most patients were male (57.1% and 71.4% in the control group and in the MI one, respectively; *p* = 0.4302). There were no significant differences between the two groups in terms of age (70.9 ± 10.8 vs. 70.0 ± 15.4 years; *p* = 0.8550). In the group of infarcted patients, five of the patients (35.7%) died in the 48 h after MI, three of the patients (21.4%) died in the week after of the ischemic event, and 42.8% of the patients (six of the patients) survived more than 2 weeks after the MI. The main cause of death in the control group was cancer in 50% of the patients. In addition, 31.4% died of non-ischemic cardiovascular disease, three of them (31.4%) died of pulmonary disease, one died of pancreatitis, one died of appendicitis, and one died of drowning.

Figure 5C illustrates the representative BiP staining patterns in cardiac autopsies from the patients with MI and those of control patients. BiP staining was observed in the cytoplasm of cardiac cells and was over-expressed in all patients that suffered an MI, except for two patients who showed minimal expression compared with those of the control group (Figure 5C,D). The hearts from patients that suffered MI not only showed BiP overexpression but also high collagen content levels compared with the controls, except for one who died in the next two hours after suffering the event (Figure 5E,F). The BiP expression and collagen content were associated (r = 0.397; *p* = 0.0329), indicating the possible role of ER stress in the development of cardiac fibrosis.

## 4. Discussion

Our study shows that the administration of the mitochondrial antioxidant MitoQ prevents the development of cardiac fibrosis and the increase in collagen I, TGF-β, and CTGF synthesis in rats with MI. In addition, circulating MPO levels, a marker of oxidative stress, were associated with levels of myocardial ECV, a surrogate marker of interstitial fibrosis, in patients that had suffered their first MI. This suggests a role of oxidative stress in the remodeling triggered by myocardial ischemia. The reduction in fibrosis in infarcted rats treated with MitoQ was accompanied by a reduction in the ER stress activation, and these beneficial effects of the mitochondrial antioxidant were reflected by an improvement in systolic and diastolic function in MI rats. Moreover, higher levels of myocardial collagen content were accompanied by the overexpression of BiP in cardiac tissues from autopsies of patients that suffered an MI compared with those who had died from different causes, supporting the participation of ER stress in the development of cardiac fibrosis in the context of myocardial ischemia. Thus, mitochondrial oxidative stress emerges as one of the factors involved in the cardiovascular remodeling associated with myocardial ischemia through the activation of ER stress.

Myocardial fibrosis is a common feature shared by different insults, including myocardial ischemia, which facilitates the development of heart failure by causing contractile dysfunction and arrhythmogenicity [18]. Different studies have shown that a higher degree of fibrosis and lesion size are associated with shorter long-term survival in patients with heart failure and other cardiac pathologies [19,20]. Our data show that patients with acute MI presented with more than 40% of the myocardium occupied by fibrosis, which was accompanied by a reduction in cardiac function. Patients showed some improvement in LV structural changes after the 12-month follow-up. However, neither the ECV nor the cardiac function had significantly improved. These results confirm the relevance of myocardial fibrosis in cardiac function and make it essential to try to reduce the excessive ECM accumulation observed in MI. Twenty percent of the LV was occupied by a necrotic scar in the first days after the event, which was reduced in the 12-month follow-up and could be due to the edema that appears abruptly a few hours after reperfusion [21].

Elevated levels of MPO are predictors of coronary artery disease [22] and the risk of MI [23]. This predictive value is explained through its inflammatory and oxidative actions, reducing the antioxidant defenses. MPO leads to alterations in the redox balance through reductions in antioxidant levels, including those of glutathione [24]. The fact that MPO levels were associated with ECV, a surrogate marker of myocardial diffuse fibrosis [25], suggests the potential role of oxidative stress in the interstitial fibrosis after myocardial ischemia. This suggestion was confirmed in the experimental setting since treatment with a mitochondrial antioxidant was able to blunt the development of diffuse fibrosis in infarcted rats. Similarly, other preclinical studies have suggested cardioprotective actions of antioxidants, not only in the context of MI but also in the context of obesity [26,27]. However, this beneficial effect has not been clearly supported in the clinical setting, even when data implying the negative association between the oxidative stress balance and LVEF function in patients with heart failure have been reported [28].

In response to ischemia, the myocardium generates ROS, especially after reperfusion [29] with cardiac myocytes and with endothelial cells and neutrophils being the main cells involved in the ROS production through several sources, including mitochondria [30]. Oxidative stress may induce permeability transition pore (mPTP) expression and can consequently trigger mitochondrial permeability. This can amplify ROS production by damaging the surrounding mitochondria and finally cause cellular injury [31]. Our data fit into this scenario because infarcted rats showed parallel increases in oxidative stress and cyclophilin F levels, which play an essential regulatory role in pore opening [32]. In this sense, it has been described that cyclophilin F levels increase in an ROS-dependent manner [33], thereby generating mitochondrial alterations. MFN1 is involved in mitochondrial fusion and, in consequence, in mitochondrial morphology. Previous studies have observed an increase in MFN1 and alterations in its activity in MI and heart failure [34,35]. The cardiac genetic depletion of MFN1 has shown beneficial effects in MI by attenuation of Ca^2+^ overload and oxidative stress [35]. The MitoQ treatment was able to prevent the increase in cyclophilin F and also the increase in MFN1 protein levels observed in infarcted animals. This supports a reduction in this compensatory mechanism to maintain energetically effective mitochondria in order to ensure cellular homeostasis [36]. MitoQ was able to reduce diffuse fibrosis in rats with MI, which can explain the improvement in diastolic function since a reduced relaxing capability of the heart can increase its filling pressure and contribute to diastolic dysfunction. In addition, MitoQ was able to improve systolic function, as supported by improvements in LVEF, SF, and the circumferential and radial strains, the latter being more sensitive markers of LV function [37]. All these beneficial effects occurred without affecting the infarct size.

Excessive ROS production shifts their regulatory role to a deleterious one. This involves the activation of different mediators, such as TGF-β [38], which plays a central role in ECM production through mechanisms that also involve ROS: the activation of fibroblasts to myofibroblasts, with more capacity of ECM production [39] or a reduction in ECM degradation [38]. In agreement, we observed an upregulation of TGF-β and of its down-stream effector, CTGF, in rats with MI, which was prevented in animals treated with MitoQ. A similar increase in the cardiac levels of both fibrotic mediators was previously reported in this context [40]. Excessive ROS generation, a consequence of an impaired mitochondrial function, perturbs protein folding, resulting in ER stress activation [10]. This can facilitate the development of cardiac fibrosis observed in infarcted rats. This notion is based on the fact that the decrease in oxidative stress levels observed in infarcted MitoQ-treated rats was accompanied by a reduction in both cardiac ER stress activation and fibrosis. Moreover, BiP, a marker of ER stress, was associated with both total fibrosis and collagen I contents in the hearts of infarcted rats. The role of ER stress in the development of fibrosis in the context of myocardial ischemia is further supported by the fact that infarcted hearts showed higher BiP expression than those from patients who died from another cause. Furthermore, the higher BiP expression observed in the cardiac autopsies of MI patients was accompanied by higher collagen levels. In agreement with this, previous studies have reported that ER stress modulation is accompanied by a reduction in cardiac fibrosis in several models of cardiac damage [13,41]. Fibroblasts seem to be the main cell involved in BiP overexpression in infarcted hearts [42]. Our data also suggest that ATF6α could be one of the pathways involved in the ER modulation of the cardiac fibrosis observed in infarcted rats, a role reported in other pathological contexts [41,43]. In fact, we observed in cardiofibroblasts that the collagen synthesis induced by angiotensin II was accompanied by an overexpression of ATF6α, which was prevented by the ER stress inhibitor 4-phenylbutyric acid [13].

Our data also showed an overexpression of ATF4 and CHOP down-stream of the PERK pathway in the hearts of infarcted animals, which was reduced in MitoQ-treated animals. ATF4 promotes the expression of CHOP [44], which can play an important role in ER-stress-induced apoptosis in cardiac cells in different pathological contexts [45] through the suppression of the survival protein Bcl2 [46]. However, a potential role in cardiac fibrosis has also been suggested [47]. The beneficial effects of MitoQ on ER stress activation are also reflected in CALRT protein levels. CALRT is one of the Ca^2+^-binding ER chaperones that is upregulated in response to ER stress in the heart [48]. It has been reported that its expression is upregulated during ER stress activation in cardiomyocytes, thus promoting cardiomyopathy, arrhythmia, and the development of heart failure [49]. This can stimulate ECM accumulation through different mechanisms, including the modulation of TGF-β actions [50]. In addition, CALRT can mediate ER-stress-induced apoptosis and hypertrophy, which can play a role in cardiac remodeling in the context of myocardial ischemia [48,51].

Our data show that MitoQ was unable to normalize calpain 1 levels, a Ca^2+^ dependent protease present in the cytosol and mitochondria, which contributes to protein processing and degradation. Its expression is enhanced under pathological conditions associate with Ca^2+^ overload, including MI, thereby contributing to cardiac injury [52,53] during ischemic conditions [54]. Calpain 1 activation is involved in different processes that occur in response to myocardial ischemia, including cytoskeletal remodeling, cell–cell adhesion disassembly, and cardiomyocyte death [55]. Its overexpression promotes impaired mitochondrial function, apoptosis, and pathological remodeling, whereas its inhibition has been shown to promote beneficial effects in MI [52,53].

## 5. Conclusions

In summary, we have demonstrated that the cardiac functional alterations, remodeling, and fibrosis observed after MI were accompanied by oxidative stress and ER stress activation in rats. The inhibition of mitochondrial oxidative stress with MitoQ prevented all these alterations. In the clinical context, we have found an association between ECV and the plasma levels of a marker of oxidative stress, MPO. Moreover, an association between markers of ER stress and fibrosis was found in sections of infarcted hearts from patients. All these data suggest that ER stress activation can mediate, at least in part, the role of oxidative stress in the remodeling that occurs after MI by facilitating the development of diffuse cardiac fibrosis.

## 6. Limitations

Some limitations of the present study deserve to be briefly mentioned. We were unable to measure circulating BiP levels in patients that suffered their first MI. Therefore, the lack of evaluation of the possible association with myocardial fibrosis and cardiac function as well as how they follow-up one year later represents a limitation of our study. However, the samples obtained from autopsies demonstrated that cardiac BiP levels were higher in patients that had suffered an MI than those who had not. Moreover, the BiP levels were associated with myocardial fibrosis, supporting the role of ER stress in the development of myocardial fibrosis in the context of MI. Although we measured radial and circumferential strain in the experimental study, the lack of data regarding longitudinal strain (due to the lack of specific images) represents a limitation of the study since these data could help to have more comprehensive information about cardiac mechanics and function.

## Figures and Tables

**Table 1:** Cardiac function, body mass index, and circulating MPO levels in MI patients in basal conditions and 12-month follow-up.

	BASAL	FOLLOW-UP
**BMI (kg/m^2^)**	28.7 ± 3.5	28.1 ± 3.0
**IVST (mm)**	11.5 ± 0.22	10.9 ± 1.41 *
**PWT (mm)**	10.8 ± 1.08	9.8 ± 1.01 ***
**EDD (mm)**	47.8 ± 4.5	48.6 ± 6.2 *
**ESD (mm)**	33.6 ± 5.6	32.1 ± 7.9
**LVEF (%)**	57.6 ± 7.9	55.9 ± 9.2
**E/A**	0.96 ± 0.34	1.05 ± 0.45
**ECV (%)**	43.4 ± 15.9	42.7 ± 12.7
**LV Mass (g)**	99.9 ± 15.5	96.1 ± 17.9
**Infarcted Mass (g)**	20.2 ± 16.1	15.2 ± 10.4 ***
**MPO (ng/mL)**	41.0 ± 22.2	75.4 ± 56.5 **

BMI: body mass index; IVST: interventricular septum thickness; PWT: posterior wall thickness; EDD: end-diastolic diameter; EDS: end-systolic diameter; LVEF: left ventricle ejection function; E/A: E-wave/A-wave ratio; ECV: extracellular volume fraction; MPO: myeloperoxidase. * *p* < 0.05; ** *p* < 0.01 and *** *p* < 0.001 vs. basal period.

## Data Availability

Data is contained within the article or Supplementary Materials.

## Supplementary Materials

The following supporting information can be downloaded at https://www.mdpi.com/article/10.3390/antiox11071232/s1, Table S1: Body weight, systolic blood pressure (SBP), and infarct size in control rats (CT) and rats submitted to myocardial infarction treated with vehicle (MI) or with the mitochondrial antioxidant MitoQ (MI-MQ; 50 mg/kg/day); Figures S1 and S2: Mitochondrial oxidative stress mediates cardiac structural alterations in infarcted rats; Figure S3: Representative pulsed tissue Doppler image tracing obtained from the basal interventricular septum along the longitudinal axis from the left parasternal four-chamber view pulsed wave (PW) Doppler spectral display shows E-waves as well as end-diastolic A-waves of rats submitted to myocardial infarction treated with vehicle (MI) or with the mitochondrial antioxidant MitoQ (MI-MQ; 50 mg/kg/day); Figure S4: Mitochondrial oxidative stress mediates cardiac structural and functional alterations in infarcted rats; Figure S5: Original blots corresponding to (A) Figure 2; (B) Figure 3; and (C) Figure 4.
