# Supplementary material for: Mitochondrial Oxidative Stress Promotes Cardiac Remodeling in Myocardial Infarction through the Activation of Endoplasmic Reticulum Stress
Source: Antioxidants (Basel). 2022 Jun 23;11(7):1232. doi: 10.3390/antiox11071232 (PMC9311874; doi:10.3390/antiox11071232)
Supplement: Supplementary file 1 [file antioxidants-11-01232-s001.zip › antioxidants-1740424-supplementary.pdf]

antioxidants

MDPI

SUPPLEMENTARY MATERIALS

Mitochondrial Oxidative Stress Promotes Cardiac Remodelling in Myocardial Infarction Through the Activation of Endoplasmic Reticulum Stress

Francisco V Souza-Neto [1,†] Fabian Islas [2,†] Sara Jiménez-González [1], María Luaces [2], Bunty Ramchandani [3], Ana Romero-Miranda [1], Beatriz Delgado-Valero [1], Elena Roldan-Molina [4], Melchor Saiz-Pardo [5,6], Mª Ángeles Cerón-Nieto [5], Luis Ortega-Medina [4,5,6], Ernesto Martínez-Martínez [1,7,†] ,Victoria Cachofeiro [1,7,†]

[1] Departamento de Fisiología, Facultad de Medicina, Instituto de Investigación Sanitaria Gregorio Marañón (IiSGM), Universidad Complutense de Madrid, Madrid, Spain; franvasc@ucm.es (F.V.S-N); saraji02@ucm.es (S.J-G); anarom12@ucm.es (A.R-M); beadel02@ucm.es (B.D-V).
[2] Servicio de Cardiología, Instituto Cardiovascular, Hospital Clínico San Carlos, Madrid, Spain; fabianislas@gmail.com (F.I); mluace01@ucm.es (M.L).
[3] Servicio de Cirugía Cardiaca Infantil, Hospital La Paz, Madrid, Spain; bunty.r@gmail.com (B.R).
[4] Biobanco del Hospital Clínico San Carlos. Instituto de Investigación de Salud del Hospital Clínico San Carlos, Madrid, Spain; elenamilagrosa.molina@salud.madrid.org (E.R-M); luis.ortega@salud.madrid.org (L.O-M).
[5] Departamento de Patología, Hospital Clínico San Carlos; Madrid, Spain; melchor.saiz@salud.madrid.org (M.S-P), nines.ceron@gmail.com (M.A.C-N).
[6] Departamento de Medicina Legal, Psiquiatría y Patología, Universidad Complutense de Madrid, Spain.
[7] Ciber de Enfermedades Cardiovasculares (CIBERCV), Instituto de Salud Carlos III, Majadahonda, Spain.
* Correspondence: ernmarti@ucm.es (E.M.-M.); vcara@ucm.es (V.C.); Tel.: +34-913941483 (E.M.-M.); +34-913941489 (V.C.).
† These authors contributed equally to this study.

This file includes:

1. supplemental table
5 supplemental figures

Table S1. Body weight, systolic blood pressure (SBP) and infarct size in control rats (CT) and rats submitted to myocardial infarction treated with vehicle (MI) or with the mitochondrial antioxidant MitoQ (MI-MQ; 50 mg/Kg/day).

	CT	MI	MI-MQ
**Body weight (g)**	389.9±9.6	394.8±9.7	380.8±9.4
**SBP (mmHg)**	128.4±2.2	132.5±1.1	128.7±1.9
**Infarct size/LV mass (%)**	-	25.2±11.7	32.1±14.9

antioxidants

MDPI

SUPPLEMENTARY MATERIALS

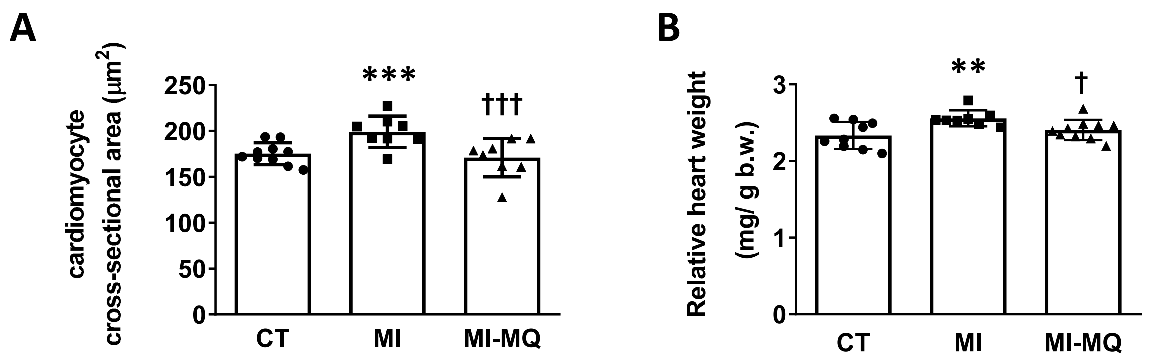

Figure S1. Mitochondrial oxidative stress mediates cardiac structural alterations in infarcted rats. (**A**) cardiomyocyte cross-sectional area; (**B**) relative heart weight expressed by body weight (b.w) in control rats (CT) and rats submitted to myocardial infarction treated with vehicle (MI) or with the mitochondrial antioxidant MitoQ (MI-MQ; 50 mg/Kg/day). Bars graphs represent the mean ± SD of 8-10 animals. **p<0.01, ***p<0.001 vs. CT group. †p<0.05, ††† p<0.001 vs. MI group.

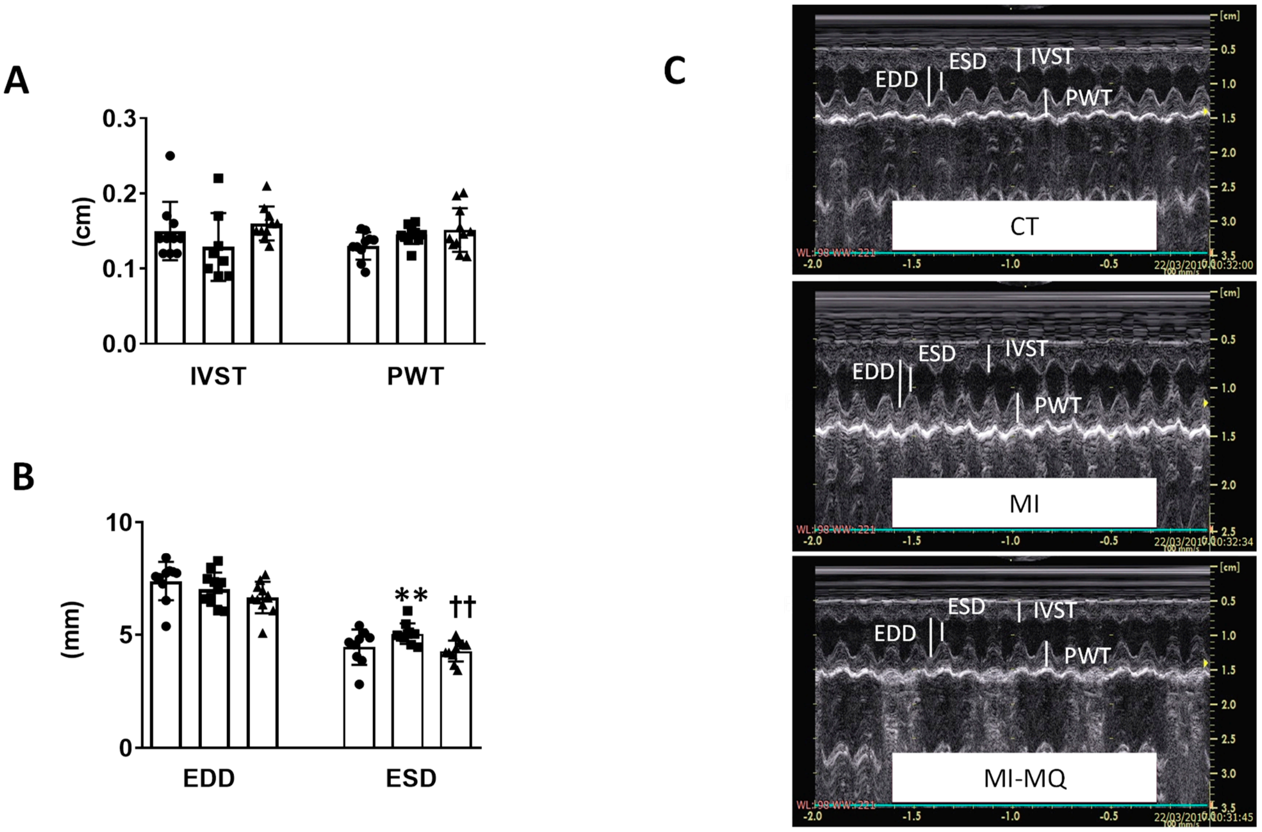

Figure S2. Mitochondrial oxidative stress mediates cardiac structural alterations in infarcted rats. (**A**) interventricular septum end-diastolic thickness (IVST) and posterior wall end-diastolic thickness (PWT); **(B)** end-diastolic diameter (EDD) and end- systolic diameter (ESD); **(C)** Representative echocardiographic images in M-mode on right parasternal short axis in control rats (CT) and rats submitted to myocardial infarction treated with vehicle (MI) or with the mitochondrial antioxidant MitoQ (MI-MQ; 50 mg/Kg/day). Bars graphs represent the mean ± SD of 8-10 animals. **p<0.01 vs. CT group; †† p<0.01 vs. MI group.

antioxidants

SUPPLEMENTARY MATERIALS

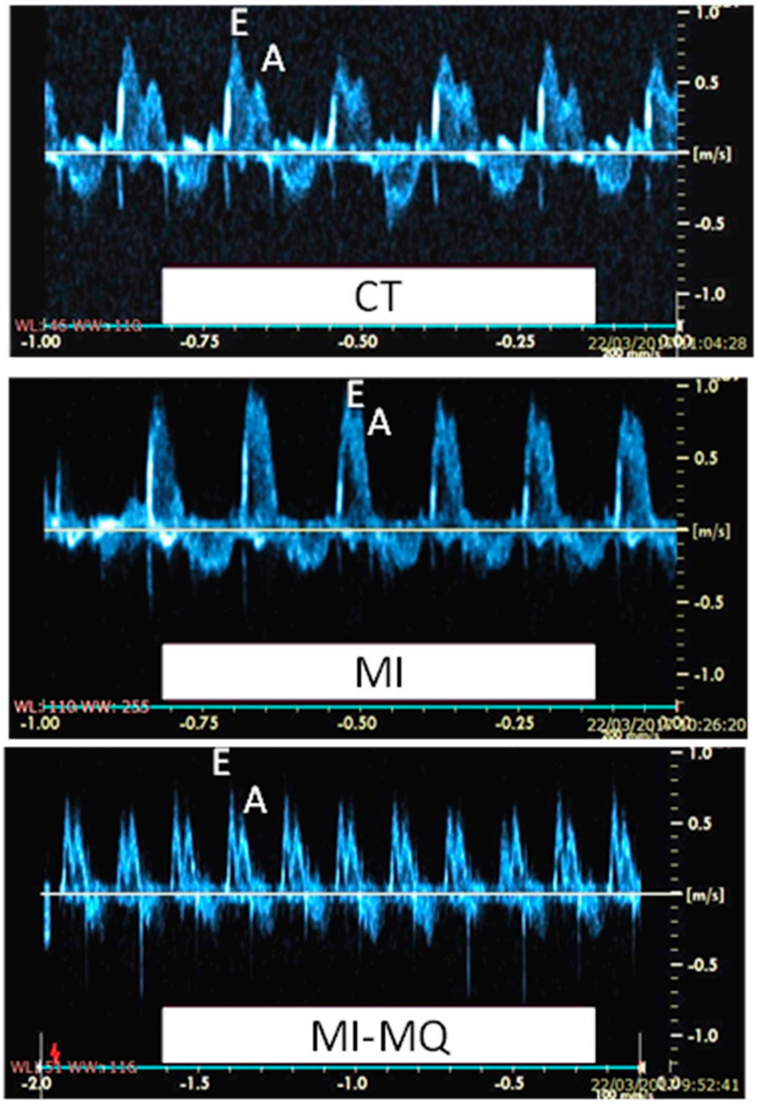

Figure S4. Representative pulsed tissue Doppler image tracing obtained from the basal interventricular septum along the longitudinal axis from the left parasternal 4-chamber view pulsed wave (PW) Doppler spectral display shows an E-wave, as well as an end-diastolic A-wave of rats submitted to myocardial infarction treated with vehicle (MI) or with the mitochondrial antioxidant MitoQ (MI-MQ; 200 µM50 mg/Kg/day).

antioxidants

MDPI

SUPPLEMENTARY MATERIALS

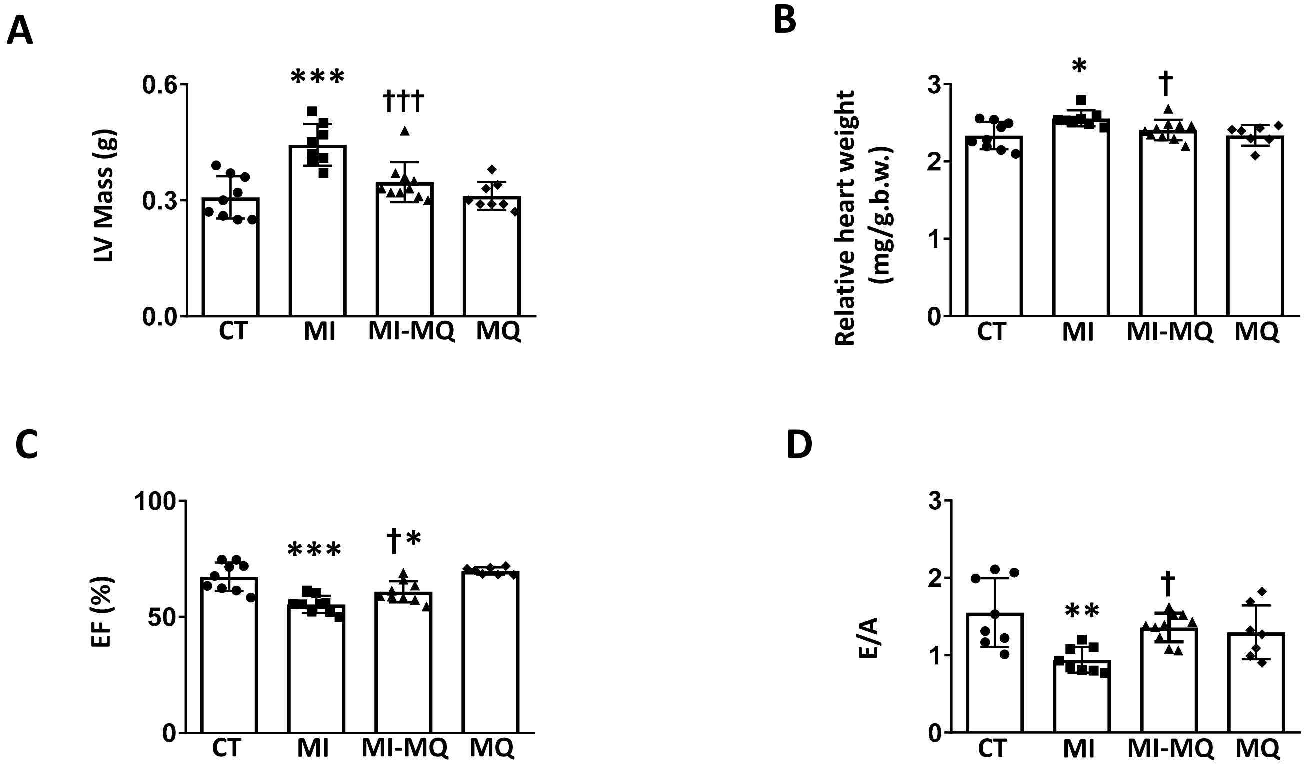

Figure S4. Mitochondrial oxidative stress mediates cardiac structural and functional alterations in infarcted rats. (A) left ventricle mass; (B) relative heart weight; (C) left ventricle ejection fraction (EF) and (D) E-wave and A- wave ratio (E/A) in control rats (CT) and rats submitted to myocardial infarction treated with vehicle (MI) or with the mitochondrial antioxidant MitoQ (MQ and MI-MQ; 50 mg/Kg/day, respectively). Bar graphs represent the mean ± SD of 7-10 animals. *$p<0.05$, **$p<0.01$, ***$p<0.001$ vs. CT group. †$p<0.05$, ††† $p<0.001$ vs. MI group.

antioxidants

MDPI

SUPPLEMENTARY MATERIALS

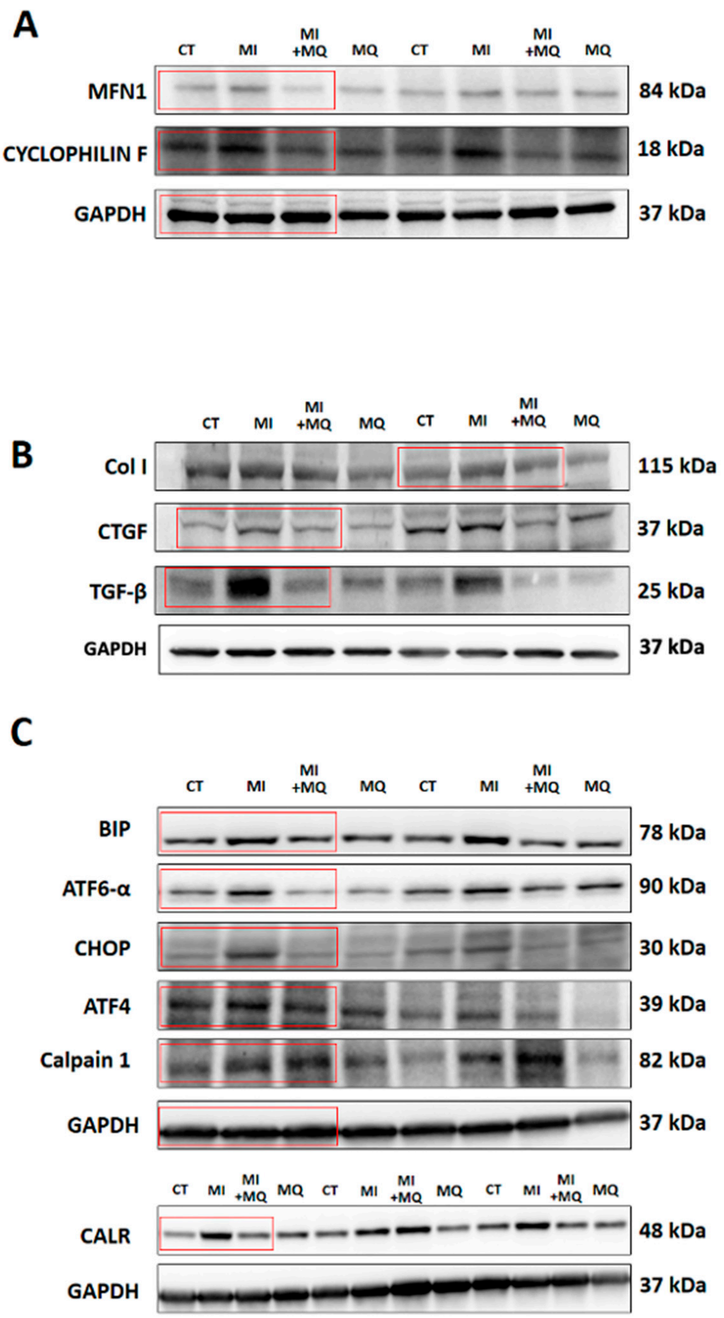

Figure S5. Original blots corresponding to (**A**) Figure 2; (**B**) Figure 3; (**C**) Figure 4.
